# Factors influencing the relationship between coaches and athletes with disabilities: a systematic review

**DOI:** 10.3389/fspor.2024.1461512

**Published:** 2024-09-16

**Authors:** Junyan Liu, Hongjun Yu, Adam Bleakney, Yih-Kuen Jan

**Affiliations:** ^1^ Department of Physical Education, Tsinghua University, Beijing, China; ^2^ Department of Health and Kinesiology, University of Illinois at Urbana-Champaign, Champaign, IL, United States

**Keywords:** adaptive sports, barriers, coaching, para-athletes, disabled

## Abstract

**Introduction:**

The relationship between coaches and athletes with disabilities is critical for enhancing athletes' performance and psychosocial well-being. This study aims to provide evidence-based recommendations for coaches, sports organizations, and policymakers dedicated to supporting athletes with disabilities.

**Methods:**

A comprehensive analysis of existing literature was conducted. Five databases were searched, including PubMed, Web of Science, SPORTDiscus, Google Scholar, and China National Knowledge Infrastructure. A total of 22 studies were included for thematic analysis.

**Results:**

This systematic review identifies key factors influencing the coach-athlete relationship in disabled sports. These factors are categorized into three main domains: professional, interpersonal, and intrapersonal. In the professional domain, effective coaching requires sport-specific skills and adaptive techniques tailored to the unique needs of athletes with disabilities. Coaches must understand the technical and tactical aspects of each parasport and adapt training plans to optimize performance and foster independence. The interpersonal domain emphasizes communication strategies and team-building practices. Effective communication involves understanding athletes' needs and adapting approaches to maximize strengths. Building rapport, managing conflict, and fostering a supportive team environment are crucial for maintaining a positive coach-athlete relationship. The intrapersonal domain highlights the importance of self-reflection and continuous learning. Coaches who engage in self-reflection and professional development better understand their behaviors and biases, enabling them to tailor coaching strategies to the specific needs of athletes with disabilities. Continuous learning is essential for remaining responsive to the evolving needs of these athletes.

**Discussion:**

This review underscores the importance of a holistic approach that integrates professional expertise, interpersonal skills, and intrapersonal awareness. By addressing these factors, it provides a foundation for developing more effective coaching strategies and supports for athletes with disabilities, ultimately enhancing their performance and well-being. Future research should explore cultural differences, the specific coaching needs of athletes with intellectual disabilities, and the impact of policies and structural barriers on the coach-athlete relationship in disabled sports.

## Background

The relationship between coaches and athletes with disabilities has garnered increasing academic attention due to the growing participation of athletes with disabilities in sports (1, 2). Understanding these interactions is essential for enhancing athletes’ performance and psychosocial well-being (3, 4). Despite the expanding body of literature, significant gaps remain in comprehensively identifying the key factors that influence these relationships, particularly within the context of disabled and Paralympic sports (5). This systematic review aims to consolidate existing research and provide evidence-based recommendations for improving coaching practices (6).

Effective coaching in disabled sports requires a blend of professional, interpersonal, and intrapersonal skills (3). These domains are critical for organizing the factors influencing the coach-athlete relationship, providing a structured approach throughout the review. The professional domain encompasses sport-specific skills and a deep understanding of disabilities, requiring adaptive communication and training strategies tailored to athletes’ needs. Key factors in this domain include coaches’ attitudes toward disability, their ability to foster an inclusive environment, and their proficiency in adaptive coaching techniques (6). Adaptive coaching, particularly within the Constraints-Led Approach (CLA), involves creating practice environments that challenge athletes to find solutions to motor problems. This method not only facilitates skill acquisition but also promotes the athlete's autonomy and decision-making abilities, essential qualities in the high-stakes world of Paralympic sports (7). The CLA positions the coach as an active participant in the learning process, emphasizing continuous interaction and dynamic adjustments based on real-time feedback, thus adding complexity to the coaching process.

The interpersonal domain focuses on the coach's ability to communicate and interact effectively with athletes and other stakeholders. Effective communication strategies, team building, and conflict management are pivotal in this domain, underscoring the importance of mutual adaptation in the coach-athlete relationship. This reciprocal process fosters a learning environment where both the coach and the athlete continually evolve, enhancing the quality of their interactions (7).

The intrapersonal domain involves the coach's self-awareness and capacity for self-reflection, including understanding their behaviors and attitudes, engaging in continuous learning, and pursuing self-improvement (3). Self-awareness is critical in adaptive coaching, as it enables coaches to adjust their methods in response to the evolving needs of athletes. This adaptability is particularly vital in managing the complex environments typical of disabled and Paralympic sports (8).

By maintaining a consistent focus on these three domains, this review provides a coherent and integrated analysis of how these domains influence coaches’ perceptions and interactions with athletes with disabilities (5). For instance, a coach's professional expertise can impact their interpersonal communication, while their self-awareness (intrapersonal domain) can influence their ability to create an inclusive environment (interpersonal domain). Addressing these gaps, particularly the integration of disability theory into coaching methodologies and the challenges related to accessibility and support, this review contributes to the development of more effective coaching strategies (6, 9).

In summary, the relationship between coaches and athletes with disabilities in disabled and Paralympic sports is crucial to the performance and well-being of these athletes. Through a comprehensive analysis of existing literature, this study provides evidence-based recommendations for coaches, sports organizations, and policymakers dedicated to supporting athletes with disabilities. This review categorizes the factors influencing the coach-athlete relationship into three key domains: professional skills, communication strategies, and self-reflection (1). These domains represent critical areas where coaches’ perceptions and interactions are most likely to impact the success and well-being of athletes with disabilities (10). By examining these specific factors, this review lays a solid foundation for developing targeted interventions and support strategies for coaches working with athletes with disabilities.

## Method

### Search strategy

This systematic review was conducted to identify relevant studies examining the factors influencing the relationship between disabled athletes and their coaches. A comprehensive search strategy was designed to cover a wide range of sources to ensure the inclusion of relevant literature. The search strategy is aligned with the Preferred Reporting Items for Systematic Reviews and Meta-Analyses (PRISMA) guidelines (11).

### Databases and search terms

The following electronic databases were comprehensively searched to identify relevant studies: PubMed, Web of Science, SPORTDiscus, Google Scholar, and China National Knowledge Infrastructure (CNKI). The search terms were developed based on key concepts related to disabled athletes, coaching, and the coach-athlete relationship. The search terms were combined using Boolean operators (AND, OR) to ensure a systematic search. The specific search strategy used was: (disabled athletes[Title/Abstract] OR para-athletes[Title/Abstract] OR disabilities[Title/Abstract] OR Paralympic Athletes[Title/Abstract] OR Wheelchair Athletes[Title/Abstract] OR Intellectual Impairments[Title/Abstract] OR Mobility Impairments[Title/Abstract] OR Special Athletes[Title/Abstract] OR Sportspeople with Disabilities[Title/Abstract] OR Parasport[Title/Abstract]) AND (coach-athlete relationship[Title/Abstract] OR coach[Title/Abstract]). The search strategy was applied to each database to retrieve articles that matched the inclusion criteria.

### Inclusion and exclusion criteria

The inclusion criteria for this review encompass studies that involve research on the relationship between disabled athletes and coaches, including quantitative studies, qualitative studies, case studies, and literature reviews. The population of focus is on interactions and relationships between disabled athletes and coaches. Articles published in English or Chinese were included, with no restrictions on publication date, from the inception of the database.

The exclusion criteria excluded non-academic literature such as news reports, blogs, opinion articles, and other non-academic publications. Studies not directly related to the relationship between disabled athletes and coaches were also excluded, as were duplicate publications of the same study or articles with duplicate content. Studies for which the full text was not accessible and those that did not pass peer review or had significant methodological flaws were also excluded (12).

### Data extraction

Data from the selected studies were systematically extracted using a standardized form (Table 1) that captured essential information, including the study title and authors, publication year, study design and methodology, population characteristics (e.g., types of disabilities and sports), key findings, factors influencing the coach-athlete relationship, and recommendations. To ensure a comprehensive synthesis of the data, two reviewers independently screened the titles and abstracts of all identified studies, followed by a detailed review of the full-text articles for potentially relevant studies.

**Table 1:** Summary of included articles in the review.

Studies	Country	Subjects			Sports	Disability	Method
		Gender	Age	Occupation			
Alexander et al. (1)	Canada, Norway, Sweden	Both male and female	Ranged from under 20 years old to over 40 years old	Athletes, Coaches, integrated Support team members (assistant coaches, high-performance directors, strength and conditioning coaches, mental performance consultants, sport physiologists, and physiotherapists)	Summer paralympic sports	Physical disabilities (including short stature and spinal cord injury) and neurological or neuro-developmental disabilities (such as cerebral palsy)	6 focus groups with athletes, 3 interviews with head coaches, 10 interviews with support team members
Alexander et al. (3)	Canada	Female	Various age	Paralympic athletes (active or retired)	Individual sports in the paralympics	Physical disabilities	Semi-structured interviews with 8 athletes, documents including athlete biographies, previous athletic records, and results from recent Paralympic Games
Alexander et al. (5)	Canada	25 female, 17 male	Various age groups (18–24, 25–34, 35–44, 45–54, and 55+)	Includes both mentor coaches (with a minimum of 10 years of experience) and mentee coaches (with under five years of experience in parasport coaching)	Various	Not specified	Semi-structured interviews
Alexander et al. (6)	Canada	Female	Not explicitly mentioned; participants attended multiple paralympic games	Paralympic athletes (active or recently retired)	Individual sports in the paralympics	Physical disabilities	Individual Semi-structured interviews
Allan et al. (10)	Canada, USA	Both male and female	Various ages, including young and older athletes	Athletes with disabilities	Various (sit skiing, sledge hockey, cross-country skiing, water sit skiing, etc.)	Various physical disabilities	Qualitative methods including interviews and reflective practice analysis
Allan et al. (13)	Canada, USA, UK; South Korea, Australia, Jordan, Israel, South Africa	Both male and female	Various age groups, including adolescents and adults	Athletes (including paralympic athletes), coaches, and military veterans	Wheelchair rugby, Swimming, Basketball (including wheelchair basketball), Sledge hockey, Adaptive sailing; Various physical activities for veterans	Physical disabilities (e.g., spinal cord injuries, cerebral palsy), Visual disabilities、Intellectual disabilities, Developmental disabilities	Semi-structured interviews, life history interviews focus groups、case studies using observations and interviews, reflexive conversations, cross-sectional questionnaires, longitudinal studies with questionnaires, observations and photographs
Dehghansai et al. (14)	Canada, USA and Australia	Both male and female	Various ages	Elite parasport athletes	Various parasports (wheelchair basketball, wheelchair rugby, etc.)	Spinal cord injury, Cerebral palsy, visual impairments	Surveys and interviews, observational studies
Lepage et al. (15)	Canada	Both male and female	Youth	Parasport coaches	Powerchair soccer, sledge hockey, and wheelchair basketball	Physical disabilities, including cerebral palsy and other physical impairments.	Semi-structured interviews
Martin et al. (16)	USA, UK, Canada, Jordan, Hungary, Portugal, Kenya, Malaysia	Both male and female	Various age ranges, including elite adult athletes and youth athletes	Athletes, specifically those participating in Paralympic and disability sports, and their coaches.	Paralympic swimming, wheelchair road racing, wheelchair rugby, wheelchair basketball, sledge hockey, parasailing	Physical disabilities, including spinal cord injuries, visual impairments, and other physical disabilities affecting mobility	Interview study, survey
Axtell et al. (17)	USA	Both male and female	Not specified	Wheelchair basketball coaches	Wheelchair basketball	Mobility impairments, specifically those using wheelchairs for sports.	Survey consisted of both multiple-choice and open-ended questions.
Cregan et al. (18)	Canada	Male	Not specified	Six elite-level coaches of swimmers with a physical disability, each with at least 10 years of head coaching experience.	Swimming	Physical disabilities, including athletes with various classifications such as S2–S10 and S5–S13.	Unstructured, Open-ended interviews
Cybulski S et al. (19)	Canada	Both male and female	Specific ages of the coaches were not provided, but they are described as experienced coaches	Coaches of Special Olympics athletes	Various	Athletes with intellectual disabilities	Semi-structured interviews, non-participant observations
Banack et al. (20)	Canada	61.06% men	Not specified	113 Canadian Paralympic athletes	Individual sports: 10.6% Team sports: 42.4% Coaching sports (e.g., swimming, track and field): 46.9%	Cerebral palsy: 23.0% Visual impairment: 8.0% Amputee: 16.8% Spinal cord injury: 44.2% Les autres (e.g., spina bifida, multiple sclerosis): 8.0%	Online survey Measures (Sport Climate Questionnaire and Sport Motivation Scale
Falcão et al. (21)	Canada	Male	The average age was 42.67 years	Head coaches of summer and winter Paralympic sport teams	Both individual and team sports within the Paralympic context.	Various (cerebral palsy, spinal-cord injuries, and amputations)	Semi-structured interviews.
Cybulski et al. (22)	Canada	Both male and female	over 8 years old	Athletes participating in the Special Olympics	Various sports (floor hockey, swimming, golf, athletics, curling, softball, ten-pin bowling, and Nordic skiing)	Intellectual disabilities (ID)	Semi-structured interviews, non-participant observations, and follow-up semi-structured interviews.
Banack et al. (23)	Canada	Both male and female	Adolescent and college age	Paralympic athletes	Various Paralympic sports	Physical disabilities	Online survey; sport climate questionnaire; measures of perceived autonomy; measures of competence; measures of relatedness; sport motivation scale
Domingues et al. (24)	Portugal	Both male and female	Mean 32.6 ± 13.8 years old	Athletes with Intellectual and Developmental Difficulty (IDD)	Special Olympics	Intellectual disabilities and developmental difficulties	Cross-sectional study, Questionnaires in Portuguese, Psychological Needs Exercise Scale (BPNES), Behaviour Regulation Sport Questionnaire (BRSQ), Satisfaction with Life Scale (SWLS), Positive and Negative Affect Schedule (PANAS), and Leadership Scale for Sport (LSS)
Wareham et al. (25)	Australia	Not specified	Not specified	12 coaches of elite athletes with disability	Various Paralympic sports (swimming, athletics, cycling, canoeing, triathlon, equestrian sport, and wheelchair basketball)	Vision impairment, amputation or limb deficiency, spinal cord injury, cerebral palsy.	Individual Semi-structured interviews
Hardwick et al. (26)	Belgium, Germany, England, Spain, Netherlands, Argentina	Both male and female	Ranges from 20 to 66 years old	Coaches and staff members involved in ParaHockey	ParaHockey	Intellectual impairments	Semi-structured interviews
Taylor et al. (27)	Australia	Both male and female	Not specified	Elite coaches in Paralympic sports, athletes with various impairments (physical, intellectual, visual)​	Various	Physical impairment Intellectual impairment Visual impairment	Semi-structured interviews, observations of remote coaching sessions, field notes, ethnographic research methods
Bloom et al. (28)	Various countries, including the USA, Canada, Croatia, UK, Denmark, and Russia	Both male and female	Ranges from youth athletes to masters athletes (older athletes)	Primarily athletes and coaches	Various sports (including football, basketball, gymnastics, ice hockey etc.)	Physical and sensory disabilities	Interviews and case studies, surveys and longitudinal studies
Burns et al. (29)	Various countries, including the UK, France, Iceland, Ireland, Sweden, Spain, Poland	Both male and female	Coaches: 22–72 years (mean age 49) Athletes: 18–42 years (mean age 24)	Coaches with at least one year's experience in coaching athletes with intellectual disabilities. Athletes actively competing in their sport for at least 6 months.	Various (basketball, tennis, equestrian, nordic Ski, gymnastics, cycling, judo, golf, table tennis)	Intellectual Disabilities	Semi-structured interviews

The synthesis of the data involved identifying common themes and patterns across the 22 included studies. Rather than simply listing individual study outcomes, we conducted a thematic analysis to integrate the findings into coherent categories that reflect the overarching trends in the literature. This process involved iteratively reviewing the extracted data to identify recurring themes and practices related to the coach-athlete relationship. The resulting themes were then used to structure the results section, providing a clear and organized presentation of the synthesized findings.

### Quality assessment

The methodological quality of the included studies was rigorously evaluated using the Mixed Methods Appraisal Tool (MMAT). This tool assesses critical aspects of research design and execution, including the clarity and appropriateness of research questions and methodologies, the validity and consistency of data collection instruments and procedures, the transparency and representativeness of participant selection processes, and the rigor of data analysis methods, such as the application of triangulation techniques.

Each study was meticulously evaluated against the MMAT criteria, resulting in varying quality scores. Studies that achieved a 100% score excelled in all areas, with clearly defined and justified research questions, robust and consistent data collection methods, transparent participant selection processes that ensured representativeness, and rigorous data analysis incorporating triangulation to enhance validity. These studies were methodologically robust and exhibited minimal risk of bias. Conversely, studies scoring 80% met many of the criteria but exhibited certain methodological limitations, such as less transparent participant selection processes, which could introduce selection bias, or data analysis methods that, while generally rigorous, lacked comprehensive triangulation. These limitations reduced their overall quality score, highlighting areas for methodological improvement.

This thorough quality assessment ensures that the studies included in this systematic review are methodologically robust, thereby enhancing the reliability and validity of the review's findings. By clearly articulating the reasons for the varying scores, we aim to maintain transparency in our assessment process, enabling readers to understand the relative strengths and limitations of the included studies (30).

### Data synthesis

A thematic synthesis approach was employed to integrate findings from qualitative, quantitative, and mixed-methods studies, ensuring comprehensive coverage of the topics discussed in the introduction. Key themes were identified through iterative coding and analysis, focusing on factors influencing the coach-athlete relationship in disabled and Paralympic sports. The synthesis examined themes within the professional, interpersonal, and intrapersonal domains, encompassing coaches’ expertise and skills, communication strategies, and self-awareness, respectively.

Specifically, the professional domain included themes related to technical and tactical knowledge, training methods, and adaptive coaching techniques suited to athletes’ specific needs. The interpersonal domain focused on effective communication strategies, team building, and the creation of inclusive environments, while the intrapersonal domain analyzed coaches’ self-reflection, continuous learning, and understanding of their behaviors and attitudes towards disabilities (31). Societal attitudes were explored to understand how perceptions and stigmas influence coaching behaviors and the training environment (32). Structural barriers such as accessibility to training facilities and availability of resources were examined to identify challenges and propose necessary policy interventions (33).

## Results

### Descriptive analysis

**Figure 1:**
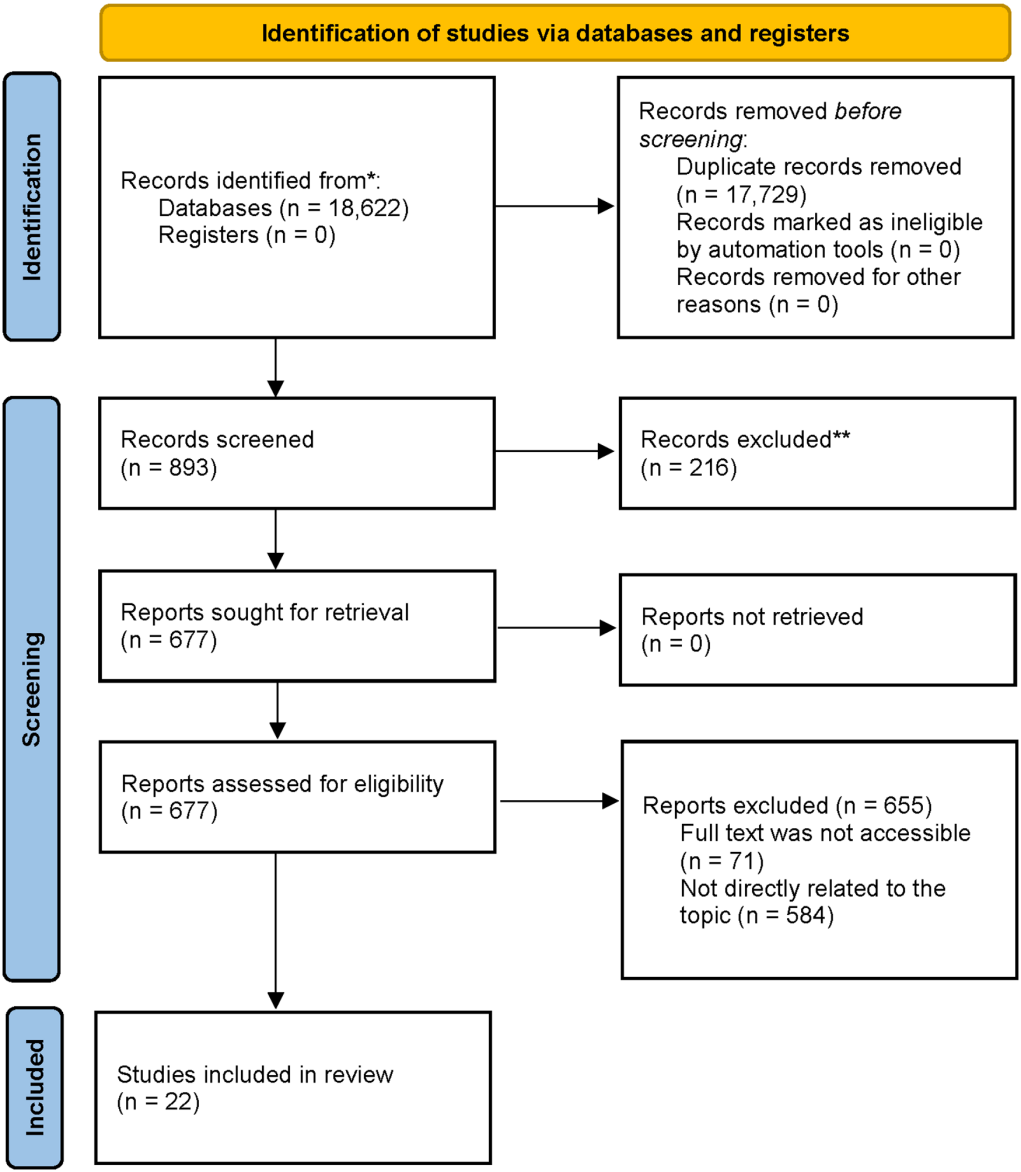
PRISMA flow diagram.

The included studies met specific inclusion criteria, focusing on the relationship between coaches and athletes with disabilities in a sports context. These peer-reviewed articles utilized various research methods, including quantitative, qualitative, and mixed-method approaches. A total of 22 studies were included in the review, ensuring a comprehensive analysis of the topic (Figure 1).

The geographical distribution of the studies provided a broad cultural perspective. The studies were conducted in Canada (*n* = 10), United States (*n* = 1), Portugal (*n* = 1), Australia (*n* = 2), various countries (*n* = 8). This variety in locations ensured that the review encompassed diverse cultural and environmental contexts. The included studies varied in design and methodology, comprising quantitative descriptive studies (*n* = 3), mixed methods studies (*n* = 3), qualitative studies (*n* = 13), and quantitative non-randomized studies (*n* = 3). This methodological diversity allowed for a thorough examination of the coach-athlete relationship from multiple perspectives.

Participants in these studies ranged from adolescents to adults, including both male and female athletes. The athletes had various types of disabilities, such as physical (*n* = 8), intellectual (*n* = 5), and multiple disabilities (*n* = 9). The studies included athletes with different levels of experience, from novices to elite competitors, providing a comprehensive overview of the coach-athlete relationship. No Chinese articles were included; all references are from English articles (*n* = 22).

### Quality assessment

A total of 22 articles were assessed for quality using the Mixed Methods Appraisal Tool (MMAT). Among these, there were 3 Quantitative Descriptive Studies, 3 Mixed Methods Studies, 13 Qualitative Studies, and 3 Quantitative Non-randomized Studies (Tables 2, 3). Of the evaluated articles, 20 were classified as high quality, meeting 100% of all quality assessment criteria. Additionally, 1 article met 80% of the quality criteria, and 1 article met 60% of the criteria. This indicates that the included studies are of generally high quality with reliable methodologies (Tables 4, 5).

**Table 2:** Quality assessment results for qualitative studies.

Studies	Types	Evaluation result							Overall MMAT score
		S1^a^	S2^b^	1.1^c^	1.2^d^	1.3^e^	1.4^f^	1,5^g^	
Alexander et al. (1)	Qualitative study	Yes	Yes	Yes	Yes	Yes	Yes	Yes	5
Alexander et al. (3)		Yes	Yes	Yes	Yes	Yes	Yes	Yes	5
Alexander et al. (6)		Yes	Yes	Yes	Yes	Yes	Yes	Yes	5
Allan et al. (10)		Yes	Yes	Yes	Yes	Yes	Yes	Yes	5
Allan et al. (13)		Yes	Yes	Yes	Yes	Yes	Yes	Yes	5
Dehghansai et al. (14, 34)		Yes	Yes	Yes	Yes	Yes	Yes	Yes	5
Lepage et al. (13)		Yes	Yes	Yes	Yes	Yes	Yes	Yes	5
Martin et al. (16)		Yes	Yes	Yes	Yes	Yes	Yes	Yes	5
Cregan et al. (18)		Yes	Yes	Yes	Yes	Yes	Yes	Yes	5
Falcão et al. (21)		Yes	Yes	Yes	Yes	Yes	Yes	Yes	5
Cybulski et al. (22)		Yes	Yes	Yes	Yes	Yes	Yes	Yes	5
Hardwick et al. (26)		Yes	Yes	Yes	Yes	Yes	Yes	Yes	5
Burns et al. (29)		Yes	Yes	Yes	Yes	Yes	Yes	Yes	5

^a^ Are there clear research questions?

^b^ Do the collected data address the research questions

^c^ Is the qualitative approach appropriate to answer the research question?

^d^ Are the qualitative data collection methods adequate to address the research question?

^e^ Are the findings adequately derived from the data?

^f^ Is the interpretation of results sufficiently substantiated by data?

^g^ Is there coherence between qualitative data sources, collection, analysis, and interpretation?

**Table 3:** Quality assessment results for mixed methods studies.

Studies	Types	Evaluation result							Overall MMAT score
		S1^a^	S2^b^	1.1^c^	1.2^d^	1.3^e^	1.4^f^	1,5^g^	
Alexander et al. (5)	Mixed methods study	Yes	Yes	Yes	Yes	Yes	Yes	Yes	5
Cybulski et al. (19)		Yes	Yes	Yes	Yes	Yes	Yes	Yes	5
Taylor et al. (27)		Yes	Yes	Yes	Yes	Yes	Yes	Yes	5

^a^ Are there clear research questions?

^b^ Do the collected data address the research questions?

^c^ Is there an adequate rationale for using a mixed methods design to address the research question?

^d^ Are the different components of the study effectively integrated to answer the research questions?

^e^ Are the results adequately brought together to answer the research questions?

^f^ Are divergences and inconsistencies between quantitative and qualitative results adequately addressed?

^g^ Do the different components of the study adhere to the quality criteria of each tradition of the methods involved?

**Table 4:** Quality assessment results for quantitative descriptive studies.

Studies	Types	Evaluation result							Overall MMAT score
		S1^a^	S2^b^	1.1^c^	1.2^d^	1.3^e^	^1.4f^	1,5^g^	
Axtell et al. (17)	Quantitative descriptive study	Yes	Yes	Yes	No	Yes	No	Yes	3
Banack et al. (20)		Yes	Yes	Yes	Yes	Yes	Yes	Yes	5
Wareham et al. (25)		Yes	Yes	Yes	Yes	Yes	Yes	Yes	5

^a^ Are there clear research questions?

^b^ Do the collected data address the research questions?

^c^ Is the sampling strategy relevant to address the quantitative research question?

^d^ Is the sample representative of the target population?

^e^ Are the measurements appropriate?

^f^ Is the risk of nonresponse bias low?

^g^ Is the statistical analysis appropriate to answer the research question?

**Table 5:** Quality Assessment results for quantitative non-randomized studies.

Studies	Types	Evaluation result							Overall MMAT score
		S1^a^	S2^b^	1.1^c^	1.2^d^	1.3^e^	1.4^f^	1,5^g^	
Banack et al. (20)	Quantitative non-randomized study	Yes	Yes	Yes	Yes	Yes	No	Yes	4
Domingues et al. (24)		Yes	Yes	Yes	Yes	Yes	Yes	Yes	5
Bloom et al. (28)		Yes	Yes	Yes	Yes	Yes	Yes	Yes	5

^a^ Are there clear research questions?

^b^ Do the collected data address the research questions?

^c^ Are the participants representative of the target population?

^d^ Are measurements appropriate regarding both the outcome and intervention (or exposure)?

^e^ Are there complete outcome data?

^f^ Are the confounders accounted for in the design and analysis?

^g^ During the study period, is the intervention administered (or exposure occurred) as intended?

### Professional domain

#### Sport-specific skills

Sport-specific skills are pivotal in optimizing athletic performance within parasports, requiring coaches to adapt their training methodologies to accommodate the unique physical and psychological characteristics of athletes with disabilities (10). Effective coaching necessitates a comprehensive understanding of the technical and tactical aspects specific to each parasport (1). For example, wheelchair basketball demands proficiency in wheelchair maneuverability, ball handling, and shooting, whereas wheelchair racing emphasizes propulsion techniques and endurance (3, 35). Coaches are responsible for developing individualized training plans that consider the specific needs of each athlete, establishing realistic performance goals, and providing constructive feedback that integrates disability-related considerations (21). This individualized approach not only enhances athletes’ confidence and motivation but also reinforces the coach-athlete relationship, thereby contributing to overall athletic success (23).

#### Adaptive coaching techniques

Adaptive coaching techniques are essential in effectively addressing the diverse needs of athletes with disabilities (1). These techniques require coaches to exhibit creativity and flexibility, ensuring that training regimens are not only effective but also inclusive. For instance, coaching swimmers with Down syndrome may involve decomposing complex movements into smaller, more manageable steps, whereas athletes with lower limb amputations in track and field may necessitate specialized training focused on prosthetic use and balance (13). Furthermore, effective communication with both athletes and their caregivers is crucial, as it fosters a supportive and inclusive training environment (1). By implementing adaptive coaching strategies, coaches can deliver personalized training that addresses the specific needs of each athlete, ultimately enhancing their performance and well-being (5, 6, 10).

### Interpersonal domain

#### Communication strategies

Effective communication is foundational to the development of strong coach-athlete relationships within parasports (3, 5). Coaches must integrate professional knowledge with advanced interpersonal skills to adequately understand and address the unique needs of athletes with disabilities (6). This necessitates an open-minded and innovative approach to communication, allowing coaches to tailor their strategies to the individual preferences and requirements of each athlete (1). Consistent, transparent communication is key to fostering trust and rapport, which are essential for athletes to feel supported and understood (10, 16). Moreover, the management of team conflicts through open dialogue and the promotion of athlete autonomy are crucial strategies that contribute to team cohesion and minimize misunderstandings (10).

#### Team building and conflict management

The construction of cohesive teams in parasports is contingent upon the establishment of an environment characterized by trust, respect, and mutual support (1). Coaches can achieve this by fostering athlete autonomy, encouraging personal ownership of roles, and modeling positive behaviors that reinforce team unity (14, 15). In the context of wheelchair basketball, for example, coaches might address conflicts arising from competitive pressures by facilitating open communication and promoting mutual support among team members (3, 5, 6). Additionally, the involvement of female coaches has been shown to enhance inclusivity and effectively address gender dynamics within teams, particularly in sports like sitting volleyball (1). Employing a democratic leadership style, wherein athletes are actively involved in decision-making processes, further strengthens team cohesion by ensuring that each member's contributions are valued and recognized (3, 6, 15).

### Intrapersonal domain

#### Self-reflection and understanding

Self-reflection is a critical component of effective coaching, especially within the context of parasports (10). Coaches must engage in continuous self-assessment to better understand and respond to the distinct needs of athletes with disabilities (1). Maintaining awareness of an athlete's overall well-being, both within and outside of sports, enables coaches to provide comprehensive support, particularly in managing the dual careers that many athletes with disabilities pursue (1). Reflexive practices, such as journaling and regular self-evaluation, are instrumental in helping coaches identify personal biases and areas for improvement, thereby allowing them to adapt their coaching strategies to better serve their athletes (3). This process of introspection is also integral to fostering trust and respect, which are vital for the success of mentor-mentee relationships (5, 25, 28, 29).

#### Continuous learning and improvement

A commitment to continuous learning is imperative for coaches operating in parasports, as it ensures they remain abreast of the latest advancements in fields such as sport psychology, nutrition, and exercise physiology (1). Coaches who prioritize ongoing professional development are better positioned to innovate within their coaching practices and adapt to the evolving needs of their athletes. Engaging in mentorship programs, attending specialized workshops, and participating in structured learning plans are effective methods for coaches to enhance their expertise (29). Moreover, practical experiences, such as collaborating with a diverse range of athletes and learning from more experienced peers, offer invaluable insights that contribute to coaching efficacy (24). By fostering a culture of continuous improvement, coaches create a dynamic and supportive environment that benefits both their own development and the performance outcomes of their athletes (6).

The summary is summarized and refined into Table 6.

**Table 6:** Summary of key findings by domain.

Domain	Skill/strategy	Description
Professional domain	Sport-specific skills	Essential for optimizing performance by tailoring training to the unique needs of athletes with disabilities. Examples include wheelchair basketball and racing
	Adaptive coaching techniques	Involves creativity and flexibility to address the diverse needs of athletes. Techniques include breaking down complex movements and specialized prosthetic training.
Interpersonal domain	Communication strategies	Crucial for building trust and rapport. Involves tailoring communication methods to individual athletes’ needs and managing team conflicts effectively
	Team Building and conflict management	Focuses on fostering team cohesion through autonomy, respect, and democratic leadership. Includes strategies for managing conflicts and the role of female coaches in promoting inclusivity
Intrapersonal domain	Self-reflection and understanding	Continuous self-assessment helps coaches better understand and meet athletes’ needs, fostering trust and respect in the coach-athlete relationship
	Continuous learning and improvement	Ongoing professional development and innovation in coaching practices are essential for adapting to evolving athlete needs and enhancing performance outcomes

## Discussions

This systematic review aimed to identify and analyze factors influencing the relationship between coaches and athletes with disabilities, focusing on professional, interpersonal, and intrapersonal domains across 22 studies from various countries. The review underscores the complexity of the coach-athlete relationship in parasports and advances our understanding by offering a nuanced analysis of these factors.

### Professional domain: sport-specific skills and adaptive techniques

While prior research has recognized the importance of technical and tactical mastery in sports, this review extends these findings by underscoring the dual necessity of addressing the unique challenges posed by various disabilities (5, 10). Effective coaching requires not only expertise in the sport but also a continuous commitment to learning and adaptation, enabling coaches to develop personalized, disability-specific approaches (22, 28). This dual focus is crucial for optimizing performance, fostering independence, and enhancing overall well-being in athletes with disabilities (21, 29, 30). By emphasizing creativity and flexibility in coaching strategies, this review offers a more holistic perspective, suggesting that these qualities are essential not just for technical proficiency but for addressing the diverse needs of athletes with disabilities across different sports contexts. This approach not only advances the current literature but also provides practical insights for coaches aiming to help athletes achieve their full potential.

### Interpersonal domain: communication and team building

Communication strategies and team-building practices are vital for establishing trust and transparency between coaches and athletes with disabilities (19). Effective communication forms the foundation of trust, creating an environment where athletes feel supported and understood, which is essential for both individual and team success (20). Trust, in turn, is integral to team cohesion, as it fosters mutual respect and support among team members (8). By promoting athlete autonomy and responsibility, coaches can cultivate a positive team environment that enhances unity and collaboration (19). However, while communication focuses on individual interactions tailored to meet the specific needs of each athlete, team building is concerned with shaping the overall team dynamic to ensure inclusivity and collective efficacy (20).

Moreover, both communication and team-building strategies are essential for conflict management, though they approach it differently (24). Transparent communication enables coaches to address misunderstandings directly with athletes, thereby preventing conflicts from escalating (36). In contrast, team-building involves structured approaches, such as fostering a democratic leadership style that encourages athlete involvement in decision-making processes (26). This not only aids in conflict resolution but also strengthens team cohesion by ensuring that all members feel their contributions are valued. The interplay between communication and team building is crucial for fostering strong coach-athlete relationships in parasports. While communication enhances individual relationships, team building aims to achieve collective harmony and unity (37). This review provides valuable insights into how these strategies can be effectively integrated, offering practical guidance for coaches to enhance both individual and team performance in parasports. Additionally, potential challenges, such as balancing individual needs with team dynamics, should be considered when applying these strategies in diverse coaching environments.

### Intrapersonal domain: self-reflection and continuous improvement

Self-reflection and continuous improvement are foundational elements in the coaching of athletes with disabilities. While previous studies have emphasized the need for self-reflective practices, this review provides a more nuanced understanding of how these practices specifically impact coaching efficacy in parasports (28). Self-reflection enhances coaches’ awareness of their athletes’ needs and plays a pivotal role in adapting coaching strategies to address the unique challenges faced by athletes with disabilities (29).

A significant contribution of this review is the synthesis of reflexive practices, such as journaling and regular self-evaluation, across various studies, demonstrating their effectiveness in improving coaching outcomes (1). While Allan et al. and Alexander et al. emphasize the importance of these practices for maintaining athlete well-being and managing dual careers, this review integrates these insights to show that reflexivity also fosters a deeper understanding of personal biases and coaching philosophies (1, 10). This comprehensive approach to self-reflection not only refines coaching strategies but also cultivates the trust and respect necessary for effective coach-athlete relationships (38). Furthermore, this review extends the current literature by exploring the role of continuous learning in developing coaching practices within parasports. Although existing studies acknowledge the necessity of ongoing education and professional development, this review elaborates on specific methods, such as mentorship programs and experiential learning, that can enhance coaching expertise (6). This synthesis highlights the dynamic interplay between formal education and practical experience, suggesting that continuous improvement involves not only acquiring new knowledge but also applying and adapting this knowledge in real-world settings (39).

Our comparative analysis underscores the unique contributions of various studies while identifying gaps in the current understanding of intrapersonal development among coaches (40). For example, while previous research has largely focused on individual components of self-reflection and continuous learning, this review synthesizes these elements to present a holistic view of how they interact to enhance coaching efficacy (8). This integrated perspective contributes to the field by offering new insights that can guide future research and practice. Additionally, future research could explore how self-reflection and continuous learning practices might differ across various disability types or coaching levels, further enriching the discourse on intrapersonal development in coaching (32).

In conclusion, this review reaffirms the importance of self-reflection and continuous improvement in coaching while advancing the discourse by providing a more comprehensive and interconnected understanding of these processes within the context of disability sports. Effective coaching is deeply rooted in the ability to continuously reflect, learn, and adapt qualities essential for meeting the complex and evolving needs of athletes with disabilities (32). This review contributes to the literature by offering a nuanced synthesis that can guide both researchers and practitioners in further exploring and enhancing intrapersonal development in coaching.

### Limitations of the existing literature

#### Cultural variability in findings

This systematic review is subject to limitations related to the potential influence of cultural variability on the findings. Although cultural factors were not explicitly examined within the scope of this review, the diversity among the included studies suggests that such factors may have a significant impact on the coach-athlete relationship in disabled sports. This variability poses challenges to the generalizability of the findings across different cultural contexts.

#### Language and database selection

A further limitation pertains to the selection of languages and databases, which have led to exclusion of pertinent studies published in non-English languages or indexed in less prominent databases. This selection bias could potentially limit the comprehensiveness of the literature reviewed, thus affecting the completeness of the conclusions drawn.

#### Heterogeneity in study designs and methodologies

The heterogeneity observed in study designs and methodologies represents a significant limitation. Variations in sample sizes, types of disabilities, sports contexts, and cultural settings across the included studies complicate the synthesis of findings. Such methodological diversity hinders the ability to draw consistent and generalizable conclusions from the data.

#### Potential overlook of organizational and societal factors

The focus of this review on professional, interpersonal, and intrapersonal domains may have inadvertently led to the neglect of important factors such as organizational policies, economic constraints, and societal attitudes toward disability sports. These factors could exert considerable influence on the coach-athlete relationship and merit further investigation.

#### Subjective nature of qualitative research

Despite the high quality of most studies as assessed by the Mixed Methods Appraisal Tool (MMAT), the inherent subjectivity of qualitative research and the methodological limitations present in some studies may compromise the robustness of the conclusions. The reliance on qualitative data introduces potential biases that warrant careful consideration.

### Future research directions

To address these limitations and advance the understanding of the coach-athlete relationship in disabled sports, future research should prioritize the following areas.

#### Investigation of cultural differences

Future studies should explore the impact of cultural differences on coaching practices and the coach-athlete relationship in disabled sports. This will facilitate the development of more context-specific recommendations that are sensitive to cultural variability.

#### Diversification of data sources

There is a need for future research to include studies published in multiple languages and sourced from a broader range of databases. This approach will enhance the comprehensiveness of the literature and ensure a more inclusive representation of global research.

#### Longitudinal study designs

Future research should employ longitudinal designs to examine the coach-athlete relationship over time. Such studies will provide deeper insights into the dynamics and evolution of this relationship, contributing to more nuanced and informed conclusions.

#### Inclusion of broader contextual factors

Future research should expand the scope to include organizational policies, economic constraints, and societal attitudes as they pertain to disability sports. Examining these broader contextual factors will yield a more holistic understanding of the variables influencing the coach-athlete relationship.

#### Development of standardized tools

The development and validation of standardized assessment frameworks and training modules should be a priority in future research. These tools will be instrumental in aiding coaches to adapt their practices to meet the diverse and unique needs of athletes with disabilities.

By addressing these limitations and focusing on these key areas, future research will be better positioned to contribute to the development of comprehensive, evidence-based coaching strategies that optimize both performance and well-being for athletes with disabilities.

## Conclusions

Our systematic review advances adaptive sports coaching by providing a comprehensive synthesis of factors influencing the coach-athlete relationship in disabled and Paralympic sports. It addresses critical gaps in the field by highlighting the dual necessity of sport-specific skills and adaptive techniques. By integrating all related factors of coaching in adaptive sports, this review proposes a new framework that enhances both performance and well-being of the athletes with disabilities, marking a departure from traditional coaching approaches in the existing literature. This review presents a nuanced guide informed by the comprehensive literature review. It emphasizes the importance of flexible adaptive strategies, effective communication, and self-reflection, providing actionable insights for coaches and policymakers, by offering an integrated understanding of the coach-athlete relationship, emphasizing the interplay between professional expertise, communication, and continuous improvement.

## Data Availability

The original contributions presented in the study are included in the article/Supplementary Material, further inquiries can be directed to the corresponding author.
